# Comparative Efficacy of Tegoprazan vs Esomeprazole/Sodium Bicarbonate for the Treatment of *Helicobacter pylori* Infection

**DOI:** 10.14309/ctg.0000000000000632

**Published:** 2023-08-10

**Authors:** Chan Hyuk Park, Jung Ho Park, Yoon Suk Jung

**Affiliations:** 1 Department of Internal Medicine, Hanyang University Guri Hospital, Hanyang University College of Medicine, Guri, Republic of Korea;; 2 Division of Gastroenterology, Department of Internal Medicine, Kangbuk Samsung Hospital, Sungkyunkwan University School of Medicine, Seoul, Republic of Korea.

**Keywords:** *Helicobacter pylori*, Eradication, p-CAB, Tegoprazan, Esomeprazole/sodium bicarbonate

## Abstract

**INTRODUCTION:**

Potassium-competitive acid blockers and proton pump inhibitors/sodium bicarbonate can rapidly increase intragastric pH. In this study, we aimed to compare the clinical outcomes of tegoprazan-based and esomeprazole/sodium bicarbonate–based triple therapies in the treatment of *Helicobacter pylori* infection.

**METHODS:**

We retrospectively reviewed the data of patients with *H. pylori* infection treated with a 14-day tegoprazan-based triple therapy or 14-day esomeprazole/sodium bicarbonate–based triple therapy. The primary end point was the *H. pylori* eradication rate with first-line treatment in an intention-to-treat analysis. Secondary end points included the eradication rate with first-line therapy in the per-protocol analysis and adverse events associated with eradication therapy.

**RESULTS:**

Of the 854 included patients, 435 were treated with tegoprazan-based therapy, and 419 received esomeprazole/sodium bicarbonate–based therapy. In the intention-to-treat population, no significant difference in eradication rate was detected between the tegoprazan-treated and esomeprazole/sodium bicarbonate–treated groups (78.6% [95% confidence interval (CI), 74.6–82.3%] vs 81.4% [95% CI, 77.4–84.9%], *P* = 0.313). The per-protocol analysis also revealed a similar eradication rate between groups (tegoprazan vs esomeprazole/sodium bicarbonate: 85.5% [95% CI, 81.8–87.5%] vs 87.8% [95% CI, 84.1–90.7%], *P* = 0.339). However, abdominal discomfort and diarrhea were more common in the esomeprazole/sodium bicarbonate–treated group than in the tegoprazan-treated group (abdominal discomfort: 1.1% vs 3.8%, *P* = 0.012; diarrhea: 9.9% vs 21.2%, *P* < 0.001).

**DISCUSSION:**

The efficacy of the esomeprazole/sodium bicarbonate–based triple therapy for *H. pylori* eradication was comparable with that of the tegoprazan-based triple therapy. However, esomeprazole/sodium bicarbonate–based therapy exhibited a higher risk of abdominal discomfort and diarrhea than tegoprazan-based therapy.

## INTRODUCTION

In 1989, Unge et al (1) proposed dual therapy, comprising a proton pump inhibitor (PPI) and amoxicillin, for eradicating *Helicobacter pylori* infection. PPIs have been an essential component of *H. pylori* eradication regimens (2). Gastric acid inhibition is crucial for increasing the *H. pylori* eradication rate by stabilizing acid-labile antibiotics in the stomach and increasing the sensitivity of *H. pylori* to antibiotics (3). Currently, PPI-based triple therapy, which consists of a PPI, amoxicillin, and clarithromycin, is the most popular eradication regimen worldwide (2,4).

There have been attempts to increase the eradication rate by suppressing gastric acid secretion more effectively using potassium-competitive acid blockers (P-CAB), which exert a faster-onset and longer-lasting acid-inhibitory effect than PPIs (5). In a Japanese randomized controlled trial comparing P-CAB–based triple therapy with PPI-based triple therapy, vonoprazan, a P-CAB, was shown to increase the *H. pylori* eradication rate by nearly 17% points when compared with a PPI (6). In a meta-analysis on the comparative efficacy of vonoprazan vs PPI, vonoprazan-based triple therapy exhibited a 1.2-times better eradication rate than PPI-based triple therapy (7). The higher eradication rate of vonoprazan-based triple therapy compared with that of PPI-based triple therapy was due to the superior efficacy of vonoprazan-based triple therapy against clarithromycin-resistant strains. Recently, a multicenter trial conducted in the United States and Europe confirmed that vonoprazan-based triple therapy exerted superior efficacy to that of PPI-based triple therapy (8). In South Korea, tegoprazan, a new P-CAB, was approved in 2021 for treating *H. pylori* infection (9,10).

Regimens comprising therapeutic agents that can facilitate the efficacy of PPI have been explored, including a combination of PPI and antacids. Most PPIs are administered as enteric-coated formulations, which are necessary to protect the acid-labile PPI from acid degradation in the stomach. Accordingly, PPIs are limited because the absorption and onset of their antisecretory effects may be delayed. To overcome these limitations, an immediate-release formulation without an enteric coating containing PPI and sodium bicarbonate was developed (11,12). Sodium bicarbonate protects uncoated PPIs from degradation by gastric acid, enabling its rapid release and absorption and improving its onset of action (11,12). For example, esomeprazole/sodium bicarbonate can rapidly increase the plasma concentration of esomeprazole through sodium bicarbonate–mediated neutralization of gastric acid and stabilization of esomeprazole in the stomach (13). In South Korea, esomeprazole/sodium bicarbonate, which has a faster onset of action than esomeprazole monotherapy, is available for acid-related disorders, such as gastroesophageal reflux disease (14).

Both P-CABs and PPIs/sodium bicarbonate are promising therapeutics for rapid symptom control in patients with gastric acid–related disorders. Tegoprazan and esomeprazole/sodium bicarbonate have been reported to increase the intragastric pH to more than 4 within 1 hour of administration (13,15). In addition, these agents may be effective in *H. pylori* eradication. However, no previous report has explored the comparative efficacies of P-CABs and PPIs/sodium bicarbonate in *H. pylori* eradication therapy. In addition, adverse events associated with PPI/sodium bicarbonate–based *H. pylori* eradication therapy are yet to be established. Therefore, in this study, we aimed to compare the efficacy and adverse events of tegoprazan and esomeprazole/sodium bicarbonate–based triple therapies in real-world settings.

## METHODS

### Study population

Adult patients (aged 19 years or older) with *H. pylori* infection treated with tegoprazan-based triple therapy or esomeprazole/sodium bicarbonate–based triple therapy between March 2021 and November 2022 at Kangbuk Samsung Hospital were eligible for study inclusion. Patients with a history of *H. pylori* eradication therapy or subtotal gastrectomy were excluded. We retrospectively reviewed data on patient demographics, symptoms, upper endoscopic findings, comorbidities, medications, adverse events, and results of *H. pylori* eradication therapy. The institutional review board of the ethics committee approved this study (Kangbuk Samsung Hospital: KBSMC 2022-11-009). Given the retrospective nature of this study, the need for informed consent was waived.

### *H. pylori* eradication therapy

The tegoprazan-based and esomeprazole/sodium bicarbonate–based eradication regimens were selected according to the clinician's preference in a real-world setting. The patients were classified into 2 groups according to the *H. pylori* eradication regimen as follows: (i) tegoprazan-based triple therapy group, twice daily doses of 50 mg of tegoprazan + 1,000 mg of amoxicillin + 500 mg of clarithromycin for 14 days; and (ii) esomeprazole/sodium bicarbonate–based triple therapy group, twice daily doses of 40/800 mg of esomeprazole/sodium bicarbonate + 1,000 mg of amoxicillin + 500 mg of clarithromycin for 14 days. Confirmation tests for successful eradication were performed at least 4 weeks after treatment. If first-line *H. pylori* eradication therapy failed, second-line eradication therapy was performed with bismuth-containing quadruple therapy as follows: (i) acid suppressant (either 50 mg of tegoprazan or 20 mg of rabeprazole) twice daily in combination with 120 mg of bismuth 4 times a day, 500 mg of metronidazole 3 times a day, and 500 mg of tetracycline 4 times a day for 14 days. The success of second-line eradication therapy was evaluated after ≥4 weeks of treatment.

### Study end point and measurements

The primary study end point was the *H. pylori* eradication rate with first-line treatment in an intention-to-treat (ITT) analysis. The secondary study end points were as follows: *H. pylori* eradication rate with first-line therapy in the per-protocol (PP) analysis, eradication rate with second-line treatment in the ITT and PP analyses, and adverse events associated with *H. pylori* eradication therapy.

One or more of the following tests were used to confirm the successful eradication of *H. pylori* infection: the ^13^C-urea breath test (Korea Otsuka Pharmaceutical Co., Ltd, Seoul, Korea) and histological evaluation with modified Giemsa staining. If any of the 2 tests were positive, *H. pylori* eradication was considered to have failed.

We visually assessed the severity of atrophic gastritis based on the Kimura-Takemoto classification as follows (16): mild (C-1 and C-2), moderate (C-3 and O-1), and severe (O-2 and O-3). Drug adherence was defined as the administration of ≥80% of prescribed medications. The severity of adverse events was assessed as mild (transient symptoms that improved spontaneously), moderate (symptoms that required management), or severe (symptoms that led to emergency visits) (17).

### Statistical analysis

Continuous variables, presented as mean ± SD, were compared using the Student *t* test. Categorical variables, presented as numbers with proportions, were compared using the Fisher exact test. Patients who did not receive sufficient medication (<80% of the prescribed medications) or were lost to follow-up were considered to have failed to achieve a cure in the ITT analysis. For PP analysis, patients with insufficient medications and those lost to follow-up were excluded. Logistic regression analysis was performed to identify factors associated with the failure of first-line *H. pylori* eradication therapy. Variables with *P* values <0.1 in the univariable logistic regression model were included as covariates in the multivariable analysis. *P* < 0.05 was deemed statistically significant. Data analyses were conducted using the R statistical software (version 4.2.2; R Foundation for Statistical Computing, Vienna, Austria).

## RESULTS

### Study population and baseline characteristics

**Figure 1.**
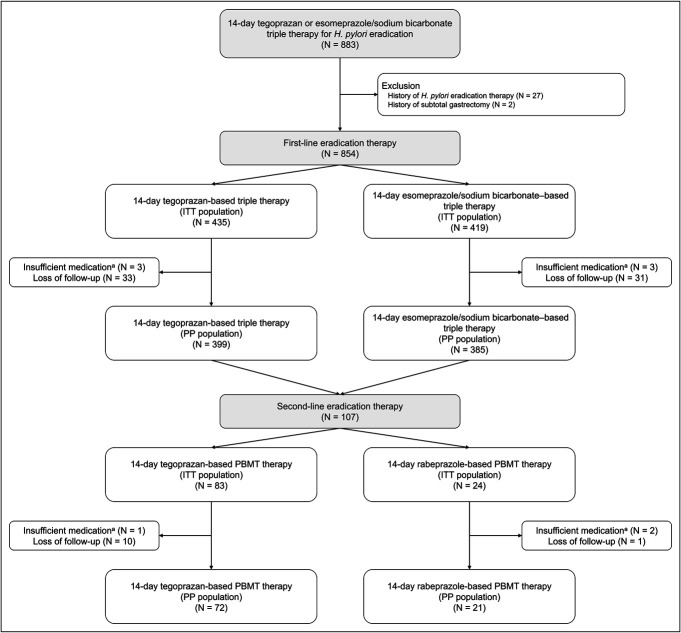
Study flow diagram. ^a^Insufficient medication is determined as administration of <80% of prescribed medications. PBMT indicates bismuth-containing quadruple therapy comprising PPI (or P-CAB), bismuth, metronidazole, and tetracycline. ITT, intention-to-treat; P-CAB, potassium-competitive acid blocker; PP, per-protocol; PPI, proton pump inhibitor.

In total, 883 patients who received tegoprazan-based or esomeprazole/sodium bicarbonate–based triple therapy for *H. pylori* infection were included in this study (Figure 1). After excluding 29 patients with a history of *H. pylori* eradication therapy or subtotal gastrectomy, 854 patients were included in the ITT analysis. Of them, 435 patients had undergone 14-day tegoprazan-based triple therapy, while 419 patients had received a 14-day esomeprazole/sodium bicarbonate–based triple therapy. After excluding 6 patients with insufficient medication and 64 who were lost to follow-up, 399 and 385 patients in the tegoprazan-treated and esomeprazole/sodium bicarbonate–treated groups, respectively, remained in the PP population. Second-line *H. pylori* eradication therapy was administered to 107 patients in whom first-line eradication therapy failed to treat the *H. pylori* infection.

Table 1 summarizes the baseline characteristics of included patients. The mean age was 56.0 ± 11.6 years in the tegoprazan group and 57.1 ± 10.9 years in the esomeprazole/sodium bicarbonate group (*P* = 0.155). The proportion of males was 55.6% and 50.1% in the tegoprazan and esomeprazole/sodium bicarbonate groups, respectively (*P* = 0.107). Hypertension was more common in the esomeprazole/sodium bicarbonate group than in the tegoprazan group (28.6% vs 21.6%, *P* = 0.018). Antithrombotic agents, particularly aspirin, were more commonly taken by patients in the esomeprazole/sodium bicarbonate group than those in the tegoprazan group (any antithrombotic agent: 12.6% vs 7.1%, *P* = 0.007; aspirin: 7.9% vs 3.4%, *P* = 0.005).

**Table 1.** Baseline characteristics of included patients

Variable	14-d tegoprazan-based triple therapy (N = 435)	14-d esomeprazole/sodium bicarbonate–based triple therapy (N = 419)	*P* value
Age, y, mean ± SD	56.0 ± 11.6	57.1 ± 10.9	0.155
Male, n (%)	242 (55.6)	210 (50.1)	0.107
BMI,^a^ kg/m^2^, mean ± SD	24.3 ± 3.3	24.2 ± 3.4	0.827
Smoking habit			0.415
Never smoker	247 (56.8)	253 (60.4)	
Former smoker	117 (26.9)	110 (26.3)	
Current smoker	71 (16.3)	56 (13.4)	
Alcohol use			0.238
Absent	235 (54.0)	218 (52.0)	
Present			
<2/wk	128 (29.4)	113 (27.0)	
≥2/wk	72 (16.6)	88 (21.0)	
Comorbidity			
Hypertension	94 (21.6)	120 (28.6)	0.018
Cardiovascular disease	17 (3.9)	22 (5.3)	0.347
Diabetes	44 (10.1)	57 (13.6)	0.114
Cerebrovascular accident	12 (2.8)	8 (1.9)	0.412
Antithrombotic agent			
Any antithrombotic agent	31 (7.1)	53 (12.6)	0.007
Aspirin	15 (3.4)	33 (7.9)	0.005
Clopidogrel	12 (2.8)	18 (4.3)	0.223
Other antiplatelet agent	4 (0.9)	4 (1.0)	> 0.999
Warfarin	0 (0.0)	1 (0.2)	0.491
NOAC	2 (0.5)	2 (0.5)	> 0.999
Others (types unknown)	2 (0.5)	1 (0.2)	> 0.999

BMI, body mass index; NOAC, nonvitamin K–dependent oral anticoagulant; N/A, not applicable.

a BMI is missing in 1 patient in the triple therapy group.

Table 2 summarizes baseline patient symptoms and endoscopic findings. The most common indication for *H. pylori* eradication was *H. pylori*-associated gastritis in both groups (tegoprazan vs esomeprazole/sodium bicarbonate: 83.4% vs 89.5%). Atrophic gastritis was more common in the esomeprazole/sodium bicarbonate group than in the tegoprazan group (96.4% vs 79.3%, *P* < 0.001).

**Table 2.** Baseline symptoms and endoscopic findings

Variable	14-d tegoprazan-based triple therapy (N = 435)	14-d esomeprazole/sodium bicarbonate–based triple therapy (N = 419)	*P* value
Symptom			
Reflux symptom^a^	10 (2.3)	13 (3.1)	0.468
Nausea or vomiting	7 (1.6)	8 (1.9)	0.739
Abdominal discomfort	93 (21.4)	89 (21.2)	0.961
Abdominal pain	12 (2.8)	9 (2.1)	0.565
Others^b^	12 (2.8)	22 (5.3)	0.063
Indication for *H. pylori* eradication			0.033
Gastric and duodenal ulcers	2 (0.5)	1 (0.2)	
Gastric ulcer	26 (6.0)	16 (3.8)	
Duodenal ulcer	30 (6.9)	24 (5.7)	
MALT lymphoma	1 (0.2)	0 (0.0)	
EGC treated with ESD	10 (2.3)	1 (0.2)	
Gastric adenoma treated with ESD	3 (0.7)	2 (0.5)	
*H. pylori*–associated gastritis	363 (83.4)	375 (89.5)	
Nodular gastritis^c^	23 (5.3)	27 (6.4)	0.472
Atrophic gastritis^c,d^			<0.001
Absent (C-0)	90 (20.7)	15 (3.6)	
Present			
C-1	112 (25.8)	81 (19.3)	
C-2	53 (12.2)	63 (15.0)	
C-3	71 (16.4)	88 (21.0)	
O-1	47 (10.8)	99 (23.6)	
O-2	43 (9.9)	58 (13.8)	
O-3	18 (4.1)	15 (3.6)	

EGC, early gastric cancer; ESD, endoscopic submucosal dissection; MALT, mucosa-associated lymphoid tissue.

a Reflux symptoms include heartburn and acid regurgitation.

b Other symptoms include globus sensation, anorexia, and belching.

c There are 2 missing values for nodular gastritis and atrophic gastritis in the triple therapy group.

d Severity of atrophic gastritis is determined by the Kimura-Takemoto classification (15).

### Efficacy of the eradication therapy

**Figure 2.**
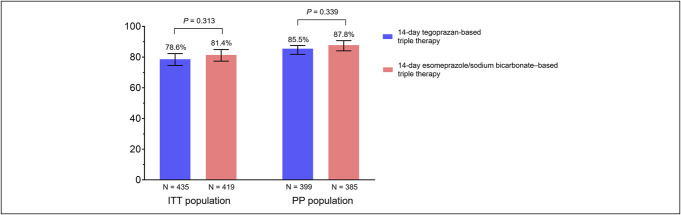
Success rate of first-line *H. pylori* eradication therapy. ITT, intention-to-treat; PP, per-protocol.

Figure 2 shows the success rate of first-line *H. pylori* eradication therapy. In the ITT population, there was no significant difference in eradication rates between the tegoprazan and esomeprazole/sodium bicarbonate groups (78.6% [95% confidence interval (CI), 74.6–82.3%] vs 81.4% [95% CI, 77.4–84.9%], *P* = 0.313). The PP analysis also revealed a similar eradication rate between the 2 groups (tegoprazan vs esomeprazole/sodium bicarbonate: 85.5% [95% CI, 81.8–87.5%] vs 87.8% [95% CI, 84.1–90.7%], *P* = 0.339).

Considering the 107 patients who received second-line therapy, 83 received 14-day tegoprazan-based bismuth-containing quadruple therapy, while 24 received 14-day rabeprazole-based bismuth-containing quadruple therapy (Figure 2). There were no significant differences between the groups in either ITT or PP analysis (tegoprazan-based quadruple vs rabeprazole-based quadruple: ITT, 84.3% [95% CI, 75.4–90.9%] vs 87.5% [95% CI, 70.3–96.4%], *P* = 0.703; PP, 95.8% [95% CI, 89.3–98.8%] vs 95.2% [95% CI, 79.8–99.5%], *P* = 0.906) (See Supplementary Figure S1, Supplementary Digital Content, http://links.lww.com/CTG/A990). We further analyzed previous eradication regimens in patients who received second-line therapy. Of the 83 patients who received tegoprazan-based quadruple therapy as second-line treatment, 52 (62.7%) received tegoprazan-based triple therapy and 31 (37.3%) received esomeprazole/sodium bicarbonate-based triple therapy as first-line treatment. Conversely, of the 24 patients who received rabeprazole-based quadruple therapy, 15 (62.5%) received esomeprazole/sodium bicarbonate-based triple therapy, while 9 (37.5%) received tegoprazan-based triple therapy.

### Treatment adherence and adverse events

Table 3 summarizes the adherence and adverse events associated with the first-line *H. pylori* eradication therapy. Adherence was >90% in both groups (tegoprazan vs esomeprazole/sodium bicarbonate: 91.7% vs 91.9%, *P* = 0.932). However, the overall adverse event rate was higher in the esomeprazole/sodium bicarbonate group than that in the tegoprazan group (39.4% vs 28.0%, *P* < 0.001). Both groups experienced mild or moderate adverse events. Specifically, abdominal discomfort and diarrhea were more common in the esomeprazole/sodium bicarbonate group than in the tegoprazan group (abdominal discomfort: 1.1% vs 3.8%, *P* = 0.012; diarrhea: 9.9% vs 21.2%, *P* < 0.001).

**Table 3.** Adherence and adverse events of first-line *H. pylori* eradication therapy

Variable	14-d tegoprazan-based triple therapy (N = 435)	14-d esomeprazole/sodium bicarbonate–based triple therapy (N = 419)	*P* value
Adherence,^a^ n (%)	399 (91.7)	385 (91.9)	0.932
Loss of follow-up	33 (7.6)	31 (7.4)	0.917
Insufficient medication	3 (0.7)	3 (0.7)	> 0.999
Adverse event,^b^ n (%)			
Any adverse event	122 (28.0)	165 (39.4)	< 0.001
Mild	119 (27.4)	162 (38.7)	
Moderate	3 (0.7)	3 (0.7)	
Severe	0 (0.0)	0 (0.0)	
General weakness	1 (0.2)	1 (0.2)	> 0.999
Dizziness	1 (0.2)	0 (0.0)	> 0.999
Headache	2 (0.5)	3 (0.7)	0.681
Myalgia	0 (0.0)	0 (0.0)	N/A
Acid regurgitation	1 (0.2)	2 (0.5)	0.618
Nausea or vomiting	14 (3.2)	16 (3.8)	0.634
Dysgeusia	53 (12.2)	62 (14.8)	0.263
Abdominal discomfort	5 (1.1)	16 (3.8)	0.012
Abdominal pain	2 (0.5)	8 (1.9)	0.060
Diarrhea	43 (9.9)	89 (21.2)	< 0.001
Constipation	3 (0.7)	2 (0.5)	> 0.999
Skin rash	8 (1.8)	2 (0.5)	0.108
Others^c^	2 (0.5)	5 (1.2)	0.279

a Adherence is determined as administration of ≥80% of prescribed medications.

b Percentage is calculated based on the ITT population.

c Other adverse events include insomnia, dry mouth, and sores on the tongue.

Adherence and adverse events associated with second-line therapy are listed in Supplementary Table S1 (see Supplementary Digital Content, http://links.lww.com/CTG/A991). Although the incidence of most adverse events did not differ between the rabeprazole-based and tegoprazan-based quadruple therapies, the tegoprazan-based quadruple therapy group exhibited a higher incidence of diarrhea than the rabeprazole-based quadruple therapy group (22.8% vs 4.2%, *P* = 0.040).

### Factors associated with failure of *H. pylori* eradication

Table 4 summarizes the logistic regression model for the failure of first-line *H. pylori* eradication therapy. Although univariable analysis revealed that sex, smoking habits, and alcohol use were associated with eradication failure, no significant associations were identified after adjusting for confounding variables. In addition, the eradication regimen (tegoprazan-based vs esomeprazole/sodium bicarbonate-based triple therapy) was not independently associated with failure of first-line *H. pylori* eradication.

**Table 4.** Factors associated with failure of first-line *H. pylori* eradication^a^

Variable	N	Failure n (%)	Univariable analysis		Multivariable analysis	
			OR (95% CI)	*P*-value	OR (95% CI)	*P*-value
Treatment duration						
14-day tegoprazan-based triple therapy	399	58 (14.5)	1		1	
14-day esomeprazole/sodium bicarbonate–based triple therapy	388	47 (12.1)	0.81 (0.54–1.23)	0.318	0.81 (0.53–1.25)	0.344
Adherence						
Adherent	783	105 (13.4)	1			
Nonadherent	4	0 (0.0)	N/A	0.999		
Age, yr						
<60	452	54 (11.9)	1			
≥60	335	51 (15.2)	1.32 (0.88–2.00)	0.182		
Sex						
Male	416	38 (9.1)	1		1	
Female	371	67 (18.1)	2.19 (1.43–3.36)	<0.001	1.62 (0.90–2.92)	0.110
BMI, kg/m^2^						
<25	474	66 (13.9)	1			
≥25	313	39 (12.5)	0.88 (0.58–1.35)	0.555		
Smoking habit						
Never smoker	463	76 (16.4)	1		1	
Former smoker	210	19 (9.0)	0.51 (0.30–0.86)	0.012	0.82 (0.42–1.61)	0.566
Current smoker	114	10 (8.8)	0.49 (0.25–0.98)	0.044	0.79 (0.34–1.82)	0.575
Alcohol use						
Absent	422	68 (16.1)	1		1	
<2/wk	222	28 (12.6)	0.75 (0.47–1.21)	0.779	0.95 (0.57–1.59)	0.854
≥2/wk	143	9 (6.3)	0.35 (0.17–0.72)	0.004	0.55 (0.25–1.20)	0.135
Comorbidity						
Hypertension	198	27 (13.6)	1.03 (0.65–1.66)	0.888		
Cardiovascular disease	38	4 (10.5)	0.76 (0.26–2.17)	0.602		
Diabetes	92	16 (17.4)	1.43 (0.80–2.57)	0.226		
Cerebrovascular accident	19	2 (10.5)	0.76 (0.17–3.34)	0.716		
Antithrombotic agent	77	9 (11.7)	0.85 (0.41–1.75)	0.654		
Symptom						
Reflux symptom^b^	19	2 (10.5)	0.76 (0.17–3.34)	0.716		
Nausea or vomiting	13	2 (15.4)	1.18 (0.26–5.42)	0.827		
Abdominal discomfort	168	30 (17.9)	1.58 (0.99–2.51)	0.054	1.42 (0.88–2.29)	0.147
Abdominal pain	18	3 (16.7)	1.31 (0.37–4.60)	0.676		
Others^c^	30	4 (13.3)	1.00 (0.34–2.92)	0.999		
Indication for *H. pylori* eradication						
Peptic ulcer	92	6 (6.5)	0.43 (0.18–1.01)	0.052	0.52 (0.22–1.25)	0.144
MALT lymphoma	1	1 (100.0)	N/A	> 0.999	N/A	> 0.999
EGC treated with ESD	11	3 (27.3)	2.30 (0.60–8.83)	0.224	2.08 (0.52–8.32)	0.303
Gastric adenoma treated with ESD	5	0 (0.0)	N/A	0.999	N/A	0.999
*H. pylori*–associated gastritis	678	95 (14.0)	1		1	
Nodular gastritis	41	8 (19.5)	1.62 (0.73–3.62)	0.237		
Atrophic gastritis^d^						
Normal (C-0)	93	12 (12.9)	1			
Mild (C-1, C-2)	283	29 (10.2)	0.77 (0.38–1.58)	0.477		
Moderate (C-3, O-1)	285	43 (15.1)	1.20 (0.60–2.39)	0.604		
Severe (O-2, O-3)	126	21 (16.7)	1.35 (0.63–2.91)	0.443		

BMI, body mass index; EGC, early gastric cancer; ESD, endoscopic submucosal dissection; MALT, mucosa-associated lymphoid tissue; OR, odds ratio; CI, confidence interval; N/A, not applicable.

a This analysis is performed on participants who received a follow-up test for *H. pylori* eradication.

b Reflux symptoms include heartburn and acid regurgitation.

c Other symptoms include insomnia, dry mouth, and sores on the tongue.

d Severity of atrophic gastritis is determined by the Kimura-Takemoto classification [14].

## DISCUSSION

P-CABs are rapid and potent gastric acid inhibitors that can overcome the limitations associated with PPIs. Therefore, in this study, among the PPI formulations comparable with P-CABs, we selected an immediate-release formulation containing esomeprazole with a strong gastric acid suppression effect and sodium bicarbonate, which enabled rapid onset of action. In this study, our findings revealed that 14-day tegoprazan-based and 14-day esomeprazole/sodium bicarbonate–based triple therapies exhibited similar efficacy in terms of *H. pylori* eradication rate. Although this study did not include an esomeprazole-based triplet regimen, we could infer the impact of sodium bicarbonate on *H. pylori* eradication therapy by reviewing the results of previous studies. In an earlier multicenter randomized controlled trial and our real-world study, tegoprazan-based triple therapy showed an eradication rate similar to that of PPI-based triple therapy (9,10). Although P-CAB typically exerts a rapid and potent acid-inhibitory effect, the dosage of P-CAB may be critical for achieving a high eradication rate. Although 50 mg of tegoprazan rapidly increases the intragastric pH > 4, its ability to maintain the intragastric pH > 6 may be weaker than that of vonoprazan (20 mg) (15). Compared with 20 mg of vonoprazan, 50 mg of tegoprazan, which is currently available in South Korea, may be insufficient to ensure a high eradication rate that exceeds PPI therapy (10). Consistently, available evidence indicates that both 50 mg of tegoprazan and the standard dose of PPI similarly affect *H. pylori* eradication. Therefore, considering the findings of previous studies and those of this study, it can be assumed that esomeprazole/sodium bicarbonate–based triple therapy may have similar efficacy to that of the PPI-based triple therapy. Accordingly, the role of sodium bicarbonate in the treatment of *H. pylori* infections remains unclear.

Considering the insignificant role of sodium bicarbonate in eradication therapy, it is believed that although sodium bicarbonate can rapidly increase the intragastric pH, it fails to maintain a sustained elevation (11,12). For example, esomeprazole/sodium bicarbonate can reduce the time required to increase the intragastric pH to ˃4 by approximately 1 hour when compared with esomeprazole alone; however, it does not increase the duration of time for which the intragastric pH remains ˃6 (13). Another study attempted to increase the eradication rate by increasing the intragastric pH with PPI before initiating eradication therapy; however, no improvement in eradication rate was observed (18). Therefore, it is unlikely that simply elevating the intragastric pH at the start of eradication therapy by using sodium bicarbonate to raise the pH rapidly will improve the eradication rate.

Additional adverse events may be a concern when using sodium bicarbonate as an adjunct to *H. pylori* eradication therapy. The risk of abdominal discomfort and diarrhea was approximately 2–3 times higher in the esomeprazole/sodium bicarbonate group than that in the tegoprazan group. Generally, the ingestion of sodium bicarbonate results in the production of CO_2_ gas owing to the dehydration of carbonic acid dissolved in water (19). Although CO_2_ is slowly produced in the stomach, it can cause abdominal bloating or cramping (19). Furthermore, sodium bicarbonate can alter the pH of the small intestine. Consequently, altered pH may result in dysbiosis in the small intestine. This may make patients taking esomeprazole/sodium bicarbonate more susceptible to antibiotic-associated diarrhea than those taking tegoprazan. Adherence was similarly high in both groups because most of the adverse events were mild and resolved spontaneously. Nevertheless, the high risk of adverse events associated with esomeprazole/sodium bicarbonate therapy weakens the rationale for treating *H. pylori* infection with esomeprazole/sodium bicarbonate.

In this study, sodium bicarbonate was not included as a second-line regimen. The second-line tegoprazan-based and rabeprazole-based quadruple therapies exhibited similar overall eradication rates. The overall adverse event rates were similar between the groups. However, diarrhea was more common in patients receiving tegoprazan-based quadruple therapy than in those receiving rabeprazole-based quadruple therapy. Considering the high risk of diarrhea with bismuth-containing quadruple therapy (10), the incidence of diarrhea with second-line rabeprazole-based quadruple therapy in this study (4.2%) was relatively low. Although the underlying cause for this inconsistency remains unclear, it could be explained by the relatively high proportion of patients who received first-line esomeprazole/sodium bicarbonate–based triple therapy in the second-line rabeprazole-based quadruple therapy group. Diarrhea may have been underestimated in these patients, given that several patients had already experienced diarrhea during the first-line treatment with sodium bicarbonate.

Our logistic regression analysis did not identify any factors associated with the failure of the first-line treatment. If we consider only the efficacy of eradication therapy, tegoprazan-based triple therapy or esomeprazole/sodium bicarbonate–based triple therapy can be selected. However, considering the risk of adverse events, tegoprazan-based triple therapy may be a better option than esomeprazole/sodium bicarbonate–based triple therapy.

However, this study is the first to investigate the efficacy of P-CAB–based triple therapy with PPI/sodium bicarbonate–based triple therapy; however, the limitations need to be addressed. First, this was a single-center retrospective study. Although our study can help understand real-world practice in patients receiving tegoprazan-based or esomeprazole/sodium bicarbonate–based triple therapy, some data, such as adverse events, particularly those related to second-line treatment, may be underestimated. Second, we could not consider the antibiotic resistance status. According to the current Korean guidelines, empirical triple therapy is allowed when the antibiotic resistance status is unknown (20). Therefore, our real-world study could only be performed in an empirical treatment setting without knowing the antibiotic resistance status. In a recent nationwide prospective study, the clarithromycin resistance rate of *H. pylori* in Korea was 17.8% (21). The high rate of clarithromycin resistance may have contributed to the poor *H. pylori* eradication rate observed in this study. Although we assumed that the clarithromycin resistance rate would be similarly high between the 2 study groups, we could not evaluate the clarithromycin resistance rate in our study because of the retrospective nature of the study design. Third, our study was conducted in Korea, where a high clarithromycin resistance rate has been recorded. Therefore, generalization of our study findings to other countries should be cautious.

Despite these limitations, our study provides a better understanding of the comparative efficacy of tegoprazan-based and esomeprazole/sodium bicarbonate–based triple therapies for the treatment of *H. pylori* infection in a real-world setting. The eradication rate did not differ between the 2 therapies; however, esomeprazole/sodium bicarbonate–based triple therapy was associated with a higher incidence of adverse events, including abdominal discomfort and diarrhea, than tegoprazan-based triple therapy.

## CONFLICTS OF INTEREST

**Guarantor of the article:** Yoon Suk Jung, MD, PhD.

**Specific author contributions:** C.H.P.: study conception and design, data acquisition, analysis, and interpretation, and manuscript drafting. J.H.P.: data acquisition and interpretation. Y.S.J.: study conception and design, data acquisition and interpretation, and overall study supervision.

**Financial support:** This work was supported by the National Research Foundation of Korea (NRF) grant funded by the Korea government (MSIP; Ministry of Science, ICT & Future Planning) (No. NRF-2021R1C1C1005728).

**Potential competing interests:** None to report.

**Study Highlights**

**WHAT IS KNOWN**

✓ Gastric acid inhibition is pivotal for the successful treatment of *Helicobacter pylori* infection.
✓ Both potassium-competitive acid blockers (e.g., tegoprazan) and proton pump inhibitors/sodium bicarbonate (e.g., esomeprazole/sodium bicarbonate) rapidly increase the intragastric pH and facilitate rapid symptom control in patients with acid-related disorders, including gastroesophageal reflux disease.

**WHAT IS NEW HERE**

✓ Tegoprazan-based triple therapy and esomeprazole/sodium bicarbonate–based triple therapy exhibited similar *H. pylori* eradication rates.
✓ Adverse events, including abdominal discomfort and diarrhea, were more common in patients who received esomeprazole/sodium bicarbonate–based triple therapy than in those who received tegoprazan-based triple therapy.

## Supplementary Material
