# Supplementary material for: Comparative Efficacy of Tegoprazan vs Esomeprazole/Sodium Bicarbonate for the Treatment of Helicobacter pylori Infection
Source: Clin Transl Gastroenterol. 2023 Aug 10;14(11):e00632. doi: 10.14309/ctg.0000000000000632 (PMC10684139; doi:10.14309/ctg.0000000000000632)
Supplement: Supplementary file 1 [file ct9-14-e00632-s001.docx]

| Table S1. Adherence and adverse events of second-line *H. pylori* eradication therapy | | | | | |
| --- | --- | --- | --- | --- | --- |
| Variable | | | 14-day tegoprazan-based PBMT therapy (N=83) | 14-day rabeprazole-based PBMT therapy (N=24) | *P*-value |
| Adherence,^a^ n (%) | | | 72 (86.7) | 21 (87.5) | > 0.999 |
|  | Loss of follow-up | | 9 (10.8) | 2 (8.3) | > 0.999 |
|  | Insufficient medication | | 2 (2.4) | 1 (4.2) | 0.537 |
| Adverse event,^b^ n (%) | | |  |  |  |
|  | Any adverse event | | 47 (56.6) | 10 (41.7) | 0.196 |
|  |  | Mild | 45 (54.2) | 9 (37.5) |  |
|  |  | Moderate | 2 (2.4) | 0 (0.0) |  |
|  |  | Severe | 0 (0.0) | 1 (4.2) |  |
|  | General weakness | | 0 (0.0) | 0 (0.0) | N/A |
|  | Dizziness | | 3 (3.6) | 0 (0.0) | > 0.999 |
|  | Headache | | 3 (3.6) | 0 (0.0) | > 0.999 |
|  | Myalgia | | 1 (1.2) | 0 (0.0) | > 0.999 |
|  | Acid regurgitation | | 1 (1.2) | 0 (0.0) | > 0.999 |
|  | Nausea or vomiting | | 23 (27.7) | 5 (20.8) | 0.500 |
|  | Dysgeusia | | 10 (12.0) | 2 (8.3) | > 0.999 |
|  | Abdominal discomfort | | 10 (12.0) | 3 (12.5) | > 0.999 |
|  | Abdominal pain | | 1 (1.2) | 0 (0.0) | >0.999 |
|  | Diarrhea | | 19 (22.8) | 1 (4.2) | 0.040 |
|  | Constipation | | 0 (0.0) | 0 (0.0) | N/A |
|  | Skin rash | | 2 (2.4) | 0 (0.0) | > 0.999 |
|  | Others^c^ | | 6 (7.2) | 1 (4.2) | > 0.999 |
| ^a^Adherence is determined as administration of ≥ 80% of prescribed medications. ^b^Percentage is calculated based on the ITT population.  ^c^Other adverse events include dry mouth and sweating. PBMT indicates bismuth-containing quadruple therapy consisting of PPI (or P-CAB), bismuth, metronidazole, and tetracycline. ITT, intention-to-treat; PP, per protocol; PPI, proton pump inhibitor; P-CAB, potassium-competitive acid blocker; N/A, not applicable. | | | | | |
